# Impact of Residual Intimal Flap Displacement Post-TEVAR on TBAD Haemodynamics in Compliant, Patient-specific CFD Simulations Informed by MRI

**DOI:** 10.1007/s10439-025-03739-6

**Published:** 2025-05-09

**Authors:** Louis Girardin, Niklas Lind, Hendrik von Tengg-Kobligk, Stavroula Balabani, Vanessa Díaz-Zuccarini

**Affiliations:** 1 Department of Mechanical Engineering, University College London, Torrington Place, London, WC1E7JE UK; 2 Welcome/ESPRC Centre for Interventional and Surgical Sciences (WEISS), 43-45 Foley Street, London, W1W7TS UK; 3 Department of Diagnostic of Interventional and Pediatric Radiology, Inselspital, 3010 Bern, Switzerland

**Keywords:** Type B aortic dissection, CFD, 4D flow MRI, Intimal flap, Compliant simulation

## Abstract

We propose a novel formulation of a moving boundary method to account for the motion of the intimal flap (IF) in a TBAD post-thoracic endovascular aortic repair using patient-specific compliant computational fluid dynamics simulations. The simulations were informed by non-invasive 4D flow MRI sequences. Predicted flow waveforms, aortic wall, and IF displacements were validated against *in vivo* 4D flow MRI and cine-MRI data. The patient-specific simulation showed that at peak systole, the dynamic interplay between high IF displacement and high transmural pressures promoted true lumen compression and false lumen expansion, whilst luminal patterns were reversed at the deceleration phase. High vorticity and swirling flow patterns were observed throughout the cardiac cycle at the primary entry tear, the descending aorta and proximal to the visceral aortic branches, correlating with high relative residence time, which could indicate an increased localised risk of aortic growth proximal to the IF. A rigid IF simulation revealed significant discrepancies in haemodynamic metrics, highlighting the potential mispredictions when using a rigid wall assumption to assess disease progression. Simulations assuming a more compliant IF highlighted potential increased risks of visceral branches malperfusion and localised aortic wall degeneration. The study underscores the necessity of patient-specific compliant IF simulations for accurate TBAD haemodynamic assessments. These insights can improve disease understanding and inform future treatment strategies.

## Introduction

Type B aortic dissection (TBAD) represents a life-threatening cardiovascular condition requiring prompt diagnosis and management to mitigate high mortality and morbidity risks. TBAD involves a primary entry tear (PET) at the descending aorta (DA), where blood seeps in the layers of the true lumen (TL), which creates the false lumen (FL); lumina are separated by the intimal flap (IF) [1]. The dissection can spread along the aorta and its branches, causing abnormal blood flow, high pressures and organ malperfusion, ultimately leading to potentially adverse outcomes. With an annual incidence affecting 1.6 in 100,000 individuals, TBAD requires close monitoring and patient-specific intervention planning to improve patient survival [2].

Surgical interventions for TBAD include thoracic endovascular aortic repair (TEVAR) and open surgery. TEVAR targets the coverage of the FL to restore normal aortic function. However, the geometric complexity of TBAD may pose challenges in achieving complete coverage, particularly when the dissection extends into branches or distally [3]. Open surgery aims to seal the PET and promote blood flow into the TL to alleviate malperfusion. To minimise coverage of collateral arteries and preserve aortic compliance, long synthetic grafts and extensive surgical replacement should be avoided [4, 5]. Despite these approaches, both surgical techniques can result in a residual dissection involving a mobile IF, complicating the restoration of optimal aortic function [6].

Whilst anatomical markers are commonly used to assess disease progression, evaluating the IF movement and the transmural pressure (TMP) can aid in predicting disease progression [7, 8]. The displacement of the IF is driven by the TMP difference between the TL and the FL, with the direction of movement determined by the sign of the TMP at any point in the cardiac cycle. There is a complex interplay between these pressure fluctuations, blood flow volume changes and the expansion and contraction of the lumina, which has been reported to promote growth and impact haemodynamics [9, 10].

Increased aortic pressures are correlated with aortic wall thickening and rigidification, leading to increased heart load [11, 12]. Moreover, pressure reflections can disrupt flow dynamics and alter wall shear stress, potentially contributing to aneurysmal growth and thrombotic events [13, 14].

4D flow MRI is an advanced imaging technique that has revolutionised the visualisation and quantification of complex flow patterns within the cardiovascular system, particularly in TBAD [15, 16]. However, several challenges still need to be addressed for widespread clinical implementation; 4D flow MRI acquisitions are costly and only available in some clinics [17]. Additionally, the large data volumes necessitate compromises in spatial and temporal resolution, typically 1–5 mm and 35–50 ms for aortic imaging [18], which limits the transient measurement of IF movements [19, 20]. Computational fluid dynamics (CFD) simulations can enhance these imaging modalities, providing a deeper understanding of the impact of IF movement on aortic haemodynamics and estimate parameters that cannot be measured directly [21]. The 2022 ACC/AHA guidelines highlight the importance of consistent imaging practices in the care of patients with aortic disease, a standard that could promote the integration of CFD as evidence for its clinical utility grows [22]. Nevertheless, many CFD studies still rely on literature-based parameters as high-quality datasets are very difficult to obtain.

Compliant models are essential for investigating the influence of IF movement on TBAD haemodynamics, with fluid–structure interaction being commonly employed. A recent fluid–structure interaction study suggested that PET size can substantially affect the TMP and haemodynamic parameters in TBAD, potentially impacting disease progression [23]. Using an idealised TBAD geometry with a constant IF thickness, the effect of IF motion on flow in acute TBAD was investigated using fluid–structure interaction [24]. They found that IF motion increased flow into the FL and predicted higher pressures than rigid wall models. Fluid–structure interaction simulations correlated near-PET haemodynamics and IF motion with potential thrombus formation [25]. Fluid–structure interaction was used to demonstrate that patient-specific IF displacements must be simulated for accurate cyclical deformation and good agreement against *in vivo* 4D flow MRI [26].

Whilst being able to provide insight on the impact of the wall and IF on TBAD haemodynamics, fluid–structure interaction-based studies often acknowledge the lack of *in vivo* tissue data to describe patient-specific material properties in the aortic wall and the IF [27]. To overcome this limitation, we have developed a moving boundary method an alternative to fluid–structure interaction that uses a deformable mesh and does not require a detailed structural model of the arterial wall to capture the interactions between the fluid and the wall. The moving boundary method is informed by patient-specific clinical images and results in substantial gains in terms of computational time [28]. In this method, the displacement of the aortic external wall and IF follows the local surface normal direction and it is linearly related to the fluid forces acting on them, with stiffness coefficients tuned based on patient-specific displacement data. However, in our previous work, the IF was modelled as a zero-thickness membrane with small displacements, which limits the accuracy of representing cross-sectional area variations [29].

In this paper, we analyse the impact of patient-specific IF displacements on the haemodynamics of a TBAD case post-TEVAR with a remaining IF with varying thickness via an improved moving boundary method. Following our previous work [30], 4D flow MRI data are exclusively utilised to inform a patient-specific compliant CFD simulation, which increases the accuracy and relevance of the findings for the individual patient, and the predictions are validated against brachial pressures, 4D flow MRI and cine-MRI. Additional simulations, including a rigid IF simulation and two others in which the IF exhibits lower degrees of stiffness leading to higher displacement are compared against the patient-specific case. Pressure and velocity magnitude distributions, the extent of the rotational flow, TMP, and wall shear stress-indices (WSS) are employed to characterise the impact of IF displacement on haemodynamics.

## Materials and Methods

### Clinical Data

**Fig. 1:**
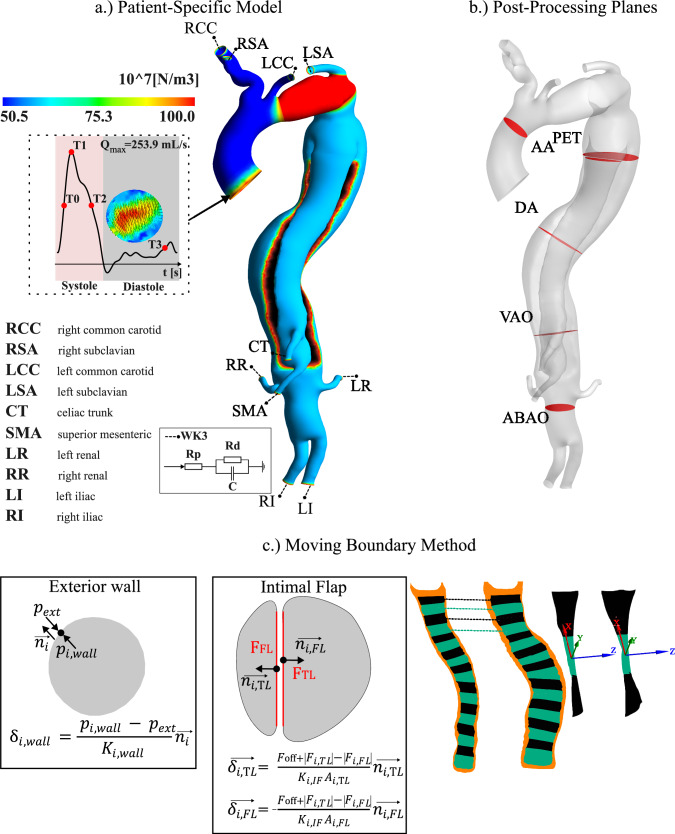
**a** Patient-specific model of the domain showing the boundary conditions used and the aortic wall stiffness. The flow curve depicts 4 distinct timepoints: T0 (mid-acceleration), T1 (peak systole), T2 (mid-deceleration), and T3 (end of diastole). **b** Planes in red are used to extract variables in the post-processing at the ascending aorta (AA), PET, descending aorta (DA), visceral aorta (VAO) and abdominal aorta (ABAO). **c** The moving boundary method is outermost boundary of the aortic wall and the IF. The rightmost part of the figure illustrates the patching technique used for the IF, showing how TL/FL pairs of patches, coloured in black and green, respectively, share the same surface normal.

A patient with chronic TBAD previously treated externally with TEVAR underwent follow-up imaging at Inselspital Bern, following an ethically approved protocol (Local Institutional Review Board ID 2019-00556), including patient consent. Brachial pressures were measured before the imaging procedures. MRI sequences were performed on a MAGNETOM Sola fit scanner (Siemens Healthineers). The imaging protocol included acquiring 4D flow MRI, two planes of cine-MRI, and T2/T1-weighted TRUFI MRI sequences, with respective pixel sizes of 2.5 × 2.5 × 2.5 ${\text{mm}}^{3}$ 1.88 × 1.88 ${\text{mm}}^{2}$ and 1 × 1 × 1 ${\text{mm}}^{3}$, to visualise the patient’s thoracic aorta. Cine-MRI planes were acquired at the ascending aorta (AA) and visceral aorta (VAO) locations (shown in Fig. 1b). 4D flow MRI and cine-MRI had a temporal resolution of 37 ms. This dissection case was selected as it represents a unique scenario where TEVAR treatment left a residual mobile IF due to partial dissection coverage.

### Segmentation and Meshing

The aortic geometry was obtained from T2/T1 TRUFI images using ScanIP (Synopsys Simpleware, USA). The segmentation involved a semi-automatic thresholding process, where a range of grayscale intensities was selected to isolate the vessel based on its unique intensity values. This allowed the creation of a mask, with smoothing performed using basic filters such as “Recursive Gaussian” and “Smart Mask” to refine the intensity at a pixel level. Further refinement involved a manual assessment using virtual brushes to address specific areas with noise or imperfections (e.g. spikes or irregularities), smoothing them by hand. This process ensured a clean, smooth surface free of folds, which is essential for accurate meshing. The geometry preparation and meshing were performed using Fluent Mesh (Ansys Fluent, USA). The IF was split longitudinally, following its curvature, forming two separate surfaces (Fig. 1c), with one surface belonging to the TL and the other the FL. Each IF surface was then divided into 10-mm segments. This division resulted in pairs of segments facing each other, alternatively shown in black and green on Fig. 1c. This ensures that the dot product of the surface normal of corresponding nodes within each pair is one or close to one [0.97–1]. This allows smooth nodal displacement within each patch pair, facilitating the application of the moving boundary method (Fig. 1c). Specifics regarding mesh element sizing, parameters applied for prism layering, and the mesh sensitivity analysis conducted to achieve the final mesh are provided in the Appendix.

The planes shown in Fig. 1b were selected to observe specific localised flow characteristics and wall displacements. For instance, the DA plane was positioned equidistantly along the vessel centerline between the PET and the VAO, ensuring it is perpendicular to the vessel and sufficiently distanced from the stent to minimise noise in proximal 4D flow MRI measurements. The AA and VAO plane were positioned so that they match the cine-MRI planes.

### Moving Boundary Method

The moving boundary method previously proposed and compared against fluid–structure interaction [29] was further developed so that it could be applied to simulate the displacement of the entire aorta, i.e. the aortic wall and the thick IF [28].

#### Aortic Wall

The local exterior wall displacement ${\updelta }_{i, wall}$ (m) is defined as follows:

1 $$\delta_{{\text{i, wall}}} \, = \,\frac{{p_{{\text{i,wall}}} \, - \,p_{{{\text{ext}}}} }}{{K_{{\text{i,wall}}} }}\overrightarrow {{n_{{\text{i}}} }},$$

where $\overrightarrow{{n}_{i}}$ is the local unit normal vector, ${\text{p}}_{\text{i,wall}}$ (Pa) is the aortic wall nodal pressure, and ${\text{p}}_{\text{ext}}$ (Pa) is the exterior pressure set as the diastolic pressure (so the expansion of the aortic wall is zero during diastole), and ${\text{K}}_{\text{i,wall}}$ ($\text{N/}{\text{m}}^{3}$) is the exterior, local wall stiffness. The stiffness is derived from the distensibility, estimated from the 4D flow MRI data and defined as the ratio between the cross-sectional relative change and the regional pulse pressure. Regions are defined using anatomical landmarks, such as the AA being delimited between the inlet and the brachiocephalic trunk, whilst pulse pressure is calculated as the difference between mean systolic and diastolic pressures (Fig. 1a; see previous works for a detailed method description [28]).

#### Intimal Flap

As described in the “Segmentation and Meshing” section, the TL and FL sections of the IF were discretised into patches along the centreline (Fig. 1c). Each patch in the TL section was paired with the nearest patch in the FL section on the opposite side of the IF. This pairing ensured that the displacement of facing IF patches is synchronised, thereby preserving the thickness of the IF.

The displacement of a pair of patches is proportional to the normal force gradient of the patches and inversely proportional to a local stiffness coefficient ${\text{K}}_{\text{i,IF}}$ ($\text{N/}{\text{m}}^{3}$) along the surface normal, such as

2 $$\overrightarrow {{\delta_{{\text{i,FL}}} }} = \frac{{F_{{{\text{off}} + }} \left| {F_{{\text{i,TL}}} } \right|\, - \,\left| {F_{{\text{i,FL}}} } \right|}}{{K_{{\text{i,IF}}} A_{{\text{i,FL}}} }}\overrightarrow {{n_{{\text{i,FL}}} }},$$

3 $$\overrightarrow {{\delta_{{\text{i,TL}}} }} \, = \, - \,\frac{{F_{{{\text{off}} + }} \left| {F_{{\text{i,TL}}} } \right|\, - \,\left| {F_{{\text{i,FL}}} } \right|}}{{K_{{\text{i,IF}}} A_{{\text{i,TL}}} }}\overrightarrow {{n_{{\text{i,TL}}} }},$$

where TL and FL denote true and false lumen, respectively, $\delta_{{\text{i,TL}}}$ and $\delta_{{\text{i,FL}}}$ (m) are the displacement, ${\text{F}}_{\text{i,TL}}$ and ${\text{F}}_{\text{i,FL}}$ (*N*) are the average forces, ${\text{A}}_{\text{i,TL}}$ and ${\text{A}}_{\text{i,FL}}$ (${\text{m}}^{2}\text{)}$ are the surface areas, and $\overrightarrow{{\text{n}}_{\text{i,TL}}}$ and $\overrightarrow{{\text{n}}_{\text{i,FL}}}$ the surface normal of each respective *i* patch TL/FL pair. ${\text{F}}_{\text{off}}$ (*N*) is the pre-stress force measured on the rigid IF simulation used to start the displacement from zero and to avoid a displacement ‘jump’ at the first simulation time step. ${\text{K}}_{\text{i,IF}}$ was iteratively tuned to match the patient-specific displacement measured on the cine-MRI.

Four IF stiffness values were then considered: a rigid one (named D0), the patient-specific stiffness (D1) derived from clinical images, and two additional cases where the stiffness is two times smaller (D2) and three times smaller (D3) than in the patient-specific case. As shown in previous work, a smoothing algorithm was used between regions of different stiffness to avoid abrupt transitions in displacement [31].

### Inlet and Outlet Boundary Conditions

Following our previous work [32], 4D flow MRI was used to extract the three-dimensional inlet velocity profile (Fig. 1a) and outlet mean flow rates using GTFlow (GyroTools LLC, Switzerland) (Table 1). MATLAB (MathWorks Inc., USA) was used to spline-interpolate the inlet flow rate to apply a 1-ms time step for the CFD simulations.

**Table 1:** Left are targeted values of inlet pressures and outlet mean flow rates—with the percentage error—against simulation results. Right are cross-sectional areas of the inlet and outlets

		Target	D0	D1 (%)	D2 (%)	D3 (%)		
Pressure (mmHg)	Diastole	84.00	82.9/1.3	82.8/1.5	82.5/1.7	82.1/2.3	Area (mm^2^)	
	Systole	113.86	113/0.7	114/− 0.1	114.9/− 0.9	115.6/− 1.5		
							Inlet	656
Mean flow rate (mL/s)	RCC	10.02	10/− 0.2	10/0.1	10/0.0	10/− 0.2	RCC	90
	RSA	6.82	6.8/− 0.3	6.8/− 0.7	6.9/− 3.7	7.1/− 1.5	RSA	30
	LCC	6.55	6.5/− 0.3	6.6/0.1	6.5/0.4	6.5/0.1	LCC	24
	LSA	10.42	10.4/1.1	10.3/0.6	10.4/0.3	10.4/0.7	LSA	39
	CT	3.51	3.5/2.3	3.4/− 1.4	3.6/0.6	3.5/1.5	CT	30
	SMA	5.72	5.7/− 2.1	5.8/− 1.7	5.8/− 2.1	5.8/−4.5	SMA	18
	LR	6.83	6.8/− 0.4	6.9/0.6	6.8/3.3	6.6/3.3	LR	25
	RR	8.35	8.4/2.8	8.1/1.2	8.3/2.7	8.1/2.1	RR	20
	LI	6.28	6.3/− 5.2	6.6/− 1.2	6.4/− 4.8	6.6/− 4.6	LI	83
	RI	5.57	5.6/2.0	5.5/0.9	5.5/2.5	5.4/2.5	RI	73

A zero-dimensional lumped parameter model of the aorta was built and tuned in 20-sim (Controllab Products, Netherlands), targeting the in vivo inlet pressures and outlet mean flow rates (Table 1). *In vivo* inlet pressures are estimated from brachial pressure measurements taken prior to MRI imaging, using an empirical formula (${\text{P}}_{\text{systole}}\text{=}{\text{0.83}{\text{P}}}_{\text{systole,brachial}}\text{+}{\text{0.15}{\text{P}}}_{\text{diastole, brachial}})$ taken from [33]. Three-element Windkessel (WK3) pressure conditions were used at the domain outlets as described in past works (Fig. 1a) [33, 34]. The WK3 parameters are provided in the Appendix.

### Computational Model

The finite-volume solver ANSYS CFX 2023R2 was utilised to solve the transient three-dimensional Navier–Stokes equations, modelling blood as an incompressible and non-Newtonian fluid with a density of 1056 kg/m^3^ and viscosity described by the Carreau–Yasuda model with empirical constants extracted from previous research [35]. The peak ${\text{Re}}_{\text{p}}$ and critical ${\text{Re}}_{\text{c}}$ Reynolds numbers were calculated as 8262 and 7407, respectively [36], indicating turbulent flow conditions, so the k-ω shear stress transport model was employed to capture turbulence effects. A low turbulence intensity of 1% was introduced to account for the laminar-turbulent transition [37]. The numerical simulations were conducted with a second-order backward Euler scheme, with convergence criteria set to a root-mean-square residual value of ${10}^{-{5}}$ for all equations within each time step. Periodic behaviour characterised by less than 1% variation in systolic and diastolic pressures between cycles was achieved after four cycles for all simulations after appropriate initialisation. The results from the final cycle were post-processed to extract relevant haemodynamic indices.

### Haemodynamics Analysis

To assess the impact of IF displacement on the TL and FL haemodynamics, TMP, vorticity, in-plane rotational flow (IRF), and WSS-driven metrics were estimated and compared for all the cases simulated.

TMP (mmHg) is the pressure difference between TL and FL:

4 $${\text{TMP}}\,{ = }\,P_{{{\text{TL}}}} \, - \,P_{{{\text{FL}}}}.$$

TMP values were extracted along the IF centreline, every 20 mm from the PET when possible for four points in the cardiac cycle: mid-acceleration (T0), peak systole (T1), mid-deceleration (T2), and end of diastole (T3).

Vorticity (1/s) was used to visualise the rotational characteristics of the blood flow. The component orthogonal to the cross-sectional planes of the aortic geometry (Fig. 1b) was calculated as follows:

5 $$\omega_{z} = \frac{\partial v}{{\partial x}}\, - \,\frac{\partial u}{{\partial y}}.$$

This vorticity was then integrated to produce the in-plane rotational flow (IRF) (m^2^/s) metric, which quantifies the strength of vorticity and has been correlated to the expansion of the FL [38]:

6 $$IRF\, = \,\iint\limits_{{T_{i} }} {\omega dS}.$$

In TBAD with residual and mobile IF, IRF measurements can provide insight into the altered haemodynamic environment and highlight potential areas contributing to the growth of the FL. The in-plane rotational flow was calculated at four points in the cardiac cycle similar to TMP, namely T0, T1, T2 and T3 at the following locations: AA, PET, DA, VAO and ABAO (see Fig. 1b).

Time averaged wall shear stress (TAWSS) (Pa), oscillatory shear index (OSI) and relative residence time (RRT) (Pa^−1^) were calculated every 5 ms [39]. These metrics are typically used to provide insights into the complex flow dynamics and potential risk areas in the aorta. Regions of low TAWSS, high OSI, and elevated RRT have been associated with adverse remodelling and potential complications in TBAD patients [40]. These metrics were computed as follows:

7 $${\text{TAWSS}}\, = \,\frac{1}{T}\mathop \smallint \limits_{0}^{T} \left| \tau \right|{\text{d}}t,$$

8 $${\text{OSI}}\, = \;0.5\left[ {1\, - \,\left( {\frac{{|\mathop \smallint \nolimits_{0}^{T} \tau {\text{d}}t|}}{{\mathop \smallint \nolimits_{0}^{T} \left| \tau \right|{\text{d}}t}}} \right)} \right],$$

9 $${\text{RRT}}\, = \,\frac{1}{{\left( {1\, - \,2.{\text{OSI}}} \right).{\text{TAWSS}}}},$$

where *T* is the cardiac cycle period (s) and *τ* the instantaneous WSS.

## Results

### Validation

#### Pressure and Flows

In the patient-specific case (D1, Table 1), target mean flow rates and pressures are simulated with maximum errors of 1.7% and 1.5%, respectively. Aortic pressures are closely matched in all other simulations. However, in D2 and D3, where the flap is more mobile, mean flow rates are simulated with errors up to 4.5% at the visceral branch outlets.

**Fig. 2:**
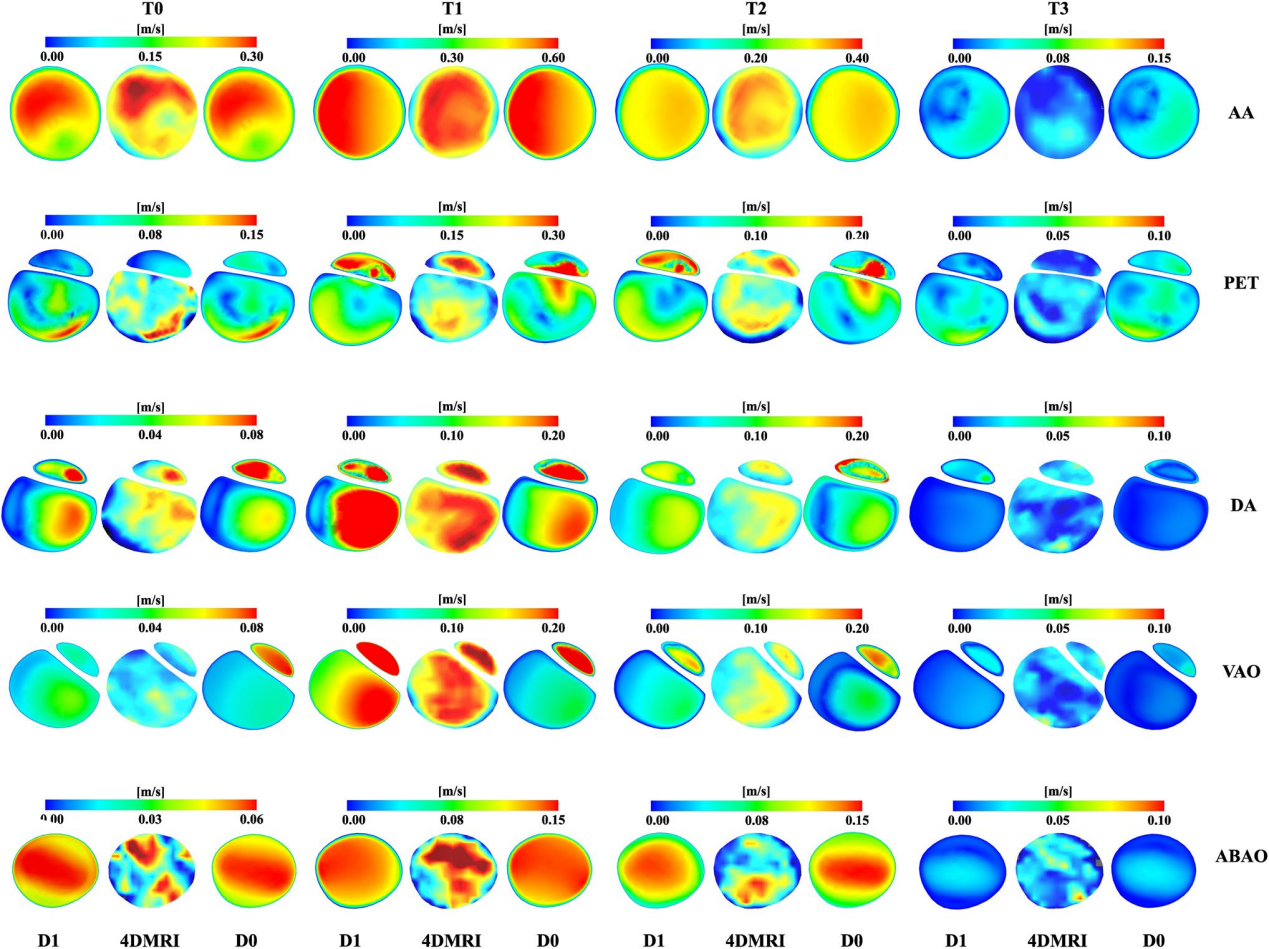
Velocity magnitude comparisons between patient-specific simulations (D1), 4D flow MRI and rigid wall simulations (D0) at mid-acceleration (T0), peak systole (T1), mid-deceleration (T2), and end of diastole (T3) in selected locations (AA, PET, DA, VAO, and ABAO).

There is a good qualitative agreement between D1 and the 4D flow MRI measurements (Fig. 2). Particularly, the velocity magnitude distributions are well matched at T0, T1, and T2, especially at the AA, PET, DA, and VAO where the patient-specific IF displacement impacts the flow. Specifically, the three-dimensional input applied at the inlet leads to good comparison with the 4D flow MRI at the AA. At the PET and DA, D1 aligns well with the 4D flow MRI at T1 and T2, especially in the TL region; in contrast, D0 predicts lower and inaccurate velocities at T1 and shows poor agreement in terms of velocity distribution with 4D flow MRI measurements at T2. Additionally, at the VAO, D0 shows excessively high FL velocity at T0 and fails to capture the velocity distribution in the TL at T1, with velocity magnitudes also significantly underpredicted.

#### Patient-Specific Wall and IF Displacements

**Fig. 3:**
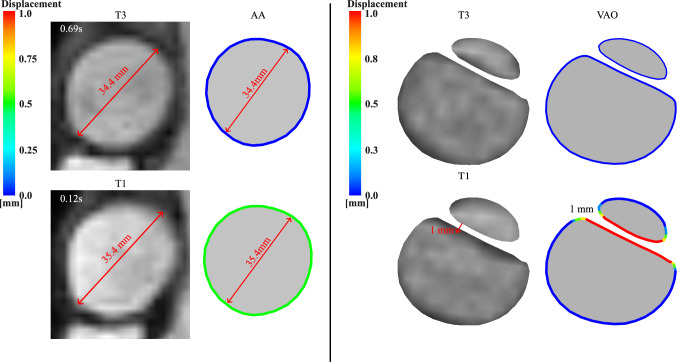
Validation of the aortic wall and IF displacement in the patient-specific case D1 against cine-MRI measurements. The left panel in each subset shows the cine-MRI and the right one shows the simulated cross-sections, with the boundary colour indicating the magnitude of the displacement. A red arrow highlights the diameter at the AA and the IF displacement.

Cine-MRI is used to validate the model by comparing the predicted displacements of the patient-specific case (D1) at the AA and VAO (Fig. 3). The comparison at the AA is made using the average diameter measured on the plane; the maximum diameter increase was 1 mm at T1. The IF displacement was measured by comparing its position at diastole (T3) against the maximum displacement at T1, which was 1 mm. The resolution of the cine-MRI does not allow for the measurement of transient displacements of the aortic wall and IF; only the peak deformations at T1 and the diastolic (T3) cross-section are measured and compared. The patient-specific simulations (D1) capture both the magnitude and pattern of the wall displacement between T1 and T3, with TL being compressed and FL expanding at T1.

### IF Displacement, TMP, and Pressure Contours

**Fig. 4:**
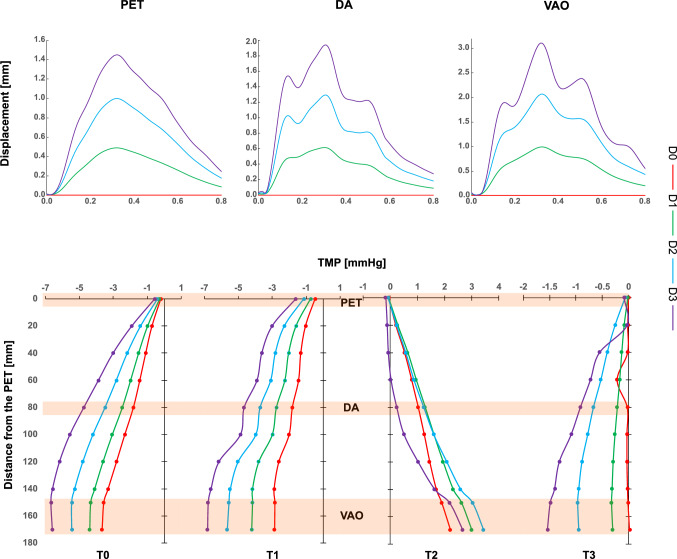
IF displacement at the PET, DA, and VAO over the cardiac cycle and TMP starting from the PET at T0, T1, T2, and T3 for all cases.

Figure 4 illustrates the displacement of the IF at the PET, DA, VAO, and TMP along the IF for each case at four different points in the cardiac cycle (T0, T1, T2, and T3). In D1, the maximum IF displacement reaches 0.5, 0.6, and 1 mm at the PET, DA, and VAO, respectively. Similarly, TMP values increase with the distance from the PET at each time point. Specifically, the TMP is negative at T0, T2, and T3 and increases linearly, with a maximum of about − 4 mmHg. At T2, the TMP is positive and correlates with the decreasing part of the displacement curve when the IF moves back towards its diastolic position and the FL is compressed.

The TMP magnitude is lower in D0 and increases with higher IF displacements. Additionally, it is worth noting that the TMP remains near 0 mmHg in D0 at T3, which inaccurately represents the TMP behaviour compared to the D1 simulation. The D0 simulation fails to capture the expected TMP increase and distribution, showing a value close to zero, except for a slight rise near the DA location. Moreover, in the most mobile IF simulation (D3), the TMP curve deviates from the trend seen in D1 at T2 and T3, relatively close to the entry tear. These differences suggest the importance of modelling the intimal flap to improve predictions of luminal pressure dynamics, which could have implications for the progression of the tear.

**Fig. 5:**
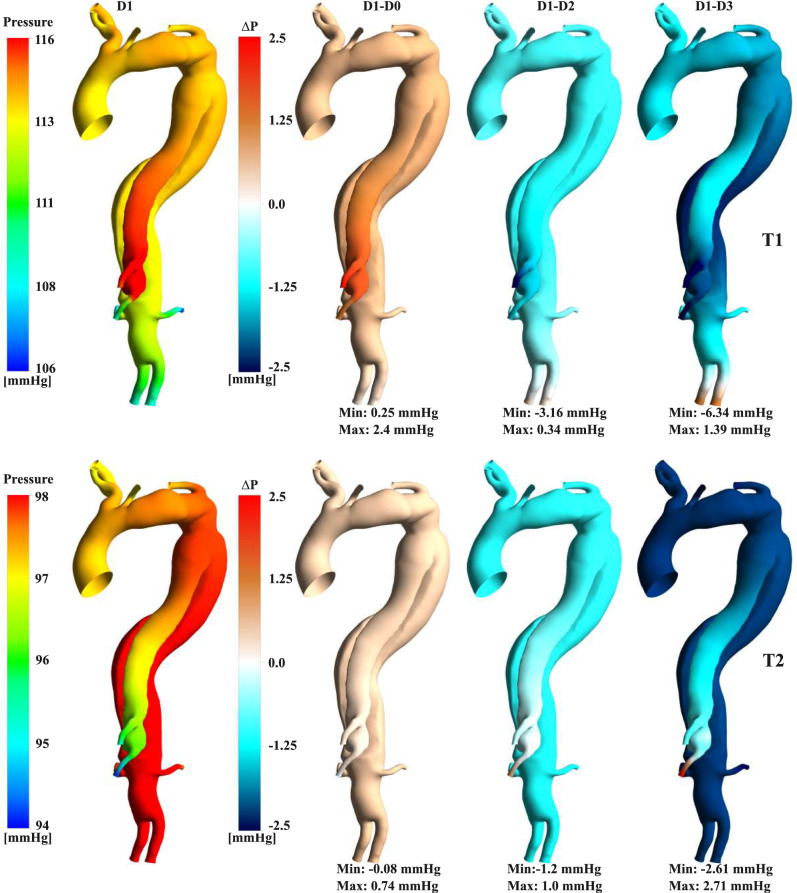
Pressure contours for D1 and point-wise difference against additional cases at T1 and T2. Maximum and minimum point-wise differences are reported below each comparison.

The pressure distribution for the patient-specific case (D1) is shown in Fig. 5 for two instants in the cardiac cycle (T1 and T2). The FL is highly pressurised at T1, with a pressure of 116 mmHg. Conversely, at T2, the TL becomes more pressurised and compressed. Comparisons against the pressure values obtained in the other cases indicate lower pressures for D0, especially at T1 in the FL, where a maximum difference of 2.4 mmHg is observed. On the contrary, the D2 and D3 cases exhibit higher pressures, reaching up to 122.34 mmHg at the visceral branches in D3, close to the location of the highest IF displacement.

### Rotational Flow Features

**Fig. 6:**
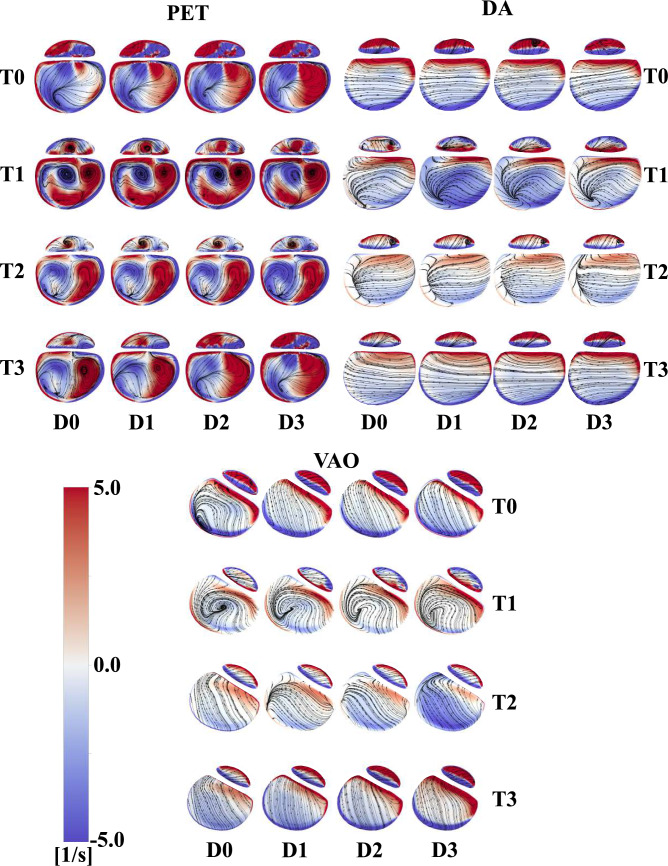
Vorticity contours and overlapping streamlines for every case at the PET, DA, and VAO at T0, T1, T2, and T3.

Vorticity patterns are analysed at selected locations (PET, DA, and VAO) (Fig. 6). In D1, vorticity patterns at PET reveal ongoing dynamic interactions between the TL and FL. In the PET region, counter-rotating vortices are clearly present in the TL, with vorticity peaking at peak systole (T2). This indicates significant rotational flow dynamics that may influence the motion of the flap separating the TL and FL. In the DA region, vorticity is less pronounced, indicating lower recirculation and a more uniform flow patterns compared to the PET region. This suggests that the flow dynamics in the DA are less likely to significantly influence flap motion. However, in the VAO region, despite overall lower vorticity values, some vortical structures are still observed at T1 and T2 for the patient-specific case (D1). These affect the flow dynamics, influencing the haemodynamic environment near the VAO and can potentially be of interest when assessing thrombotic risk.

Vorticity differences between the patient-specific case (D1) and the additional cases (D0, D2, and D3) are relatively mild at the PET. At the DA, particularly at T1—where peak IF displacement is observed—significant differences in both vorticity values and vortical structures are evident in both lumina for the patient-specific case (D1) compared to the rigid IF case (D0). Increased IF displacement (D2, D3) also leads to differences compared to the patient-specific case, especially at T1, where negative vorticity in the TL is lower and vortical structures in the FL differ. At the VAO, where the highest IF displacements occur, vortical structures differ substantially between the patient-specific case (D1) and the rigid IF case (D0) at T0, T1, and T2. Vortical structures are also different when the IF is more mobile (D2, D3) as depicted at T1 and T2, additionally, minimum and maximum vorticity values are higher.

The rotational flow characteristics observed across different regions, particularly at the FL, are reflected in the IRF values summarised in Table 2. In D1, IRF values tend to increase from T0 to T1 and then decrease during the deceleration phases (regardless of the sign), at the AA, DA, VAO, and ABAO (Table 2). High IRF magnitudes (>20 cm^2^/s) are particularly noted in the AA, PET, and VAO at T1, suggesting significant rotational flow in these regions. Conversely, IRF values at the DA remain close to zero, indicating a balance between positive and negative vorticity. At the PET, notable differences are observed between the patient-specific simulation (D1) and the rigid IF model (D0). These disparities are most evident at T1, where the IRF is underpredicted in the TL and overpredicted in the FL, which may affect prognosis related to growth and vascular remodelling. Furthermore, at the PET, the increased magnitude of the IRF suggests a higher risk of growth, particularly with greater displacement of the more mobile IF (D2 and D3). Overall, when comparing the patient-specific (D1) case against the rigid wall (D0) case, differences in vorticity patterns and vortex strength emphasise the potential influence of IF motion on flow dynamics in regions downstream of the dissection, particularly during systole (T0, T1, and T2).

**Table 2:** IRF measured at the AA and ABAO, and in both lumina at the PET, DA, and VAO for every case at T0, T1, T2, and T3

		IRF [cm^2^/s]			
		D0	D1	D2	D3
AA		34.30	34.22	34.08	33.92
PET	TL	25.72	33.92	44.56	56.37
	FL	− 13.33	− 6.01	2.22	11.20
DA	TL	− 1.11	−0.99	− 0.83	− 0.61
	FL	− 1.84	− 1.48	− 1.05	− 0.57
VAO	TL	− 19.47	− 18.15	− 15.86	− 12.47
	FL	− 0.70	− 0.52	− 0.27	0.60
ABAO		5.38	5.62	5.95	6.36

### Wall Shear Stress Indices

**Fig. 7:**
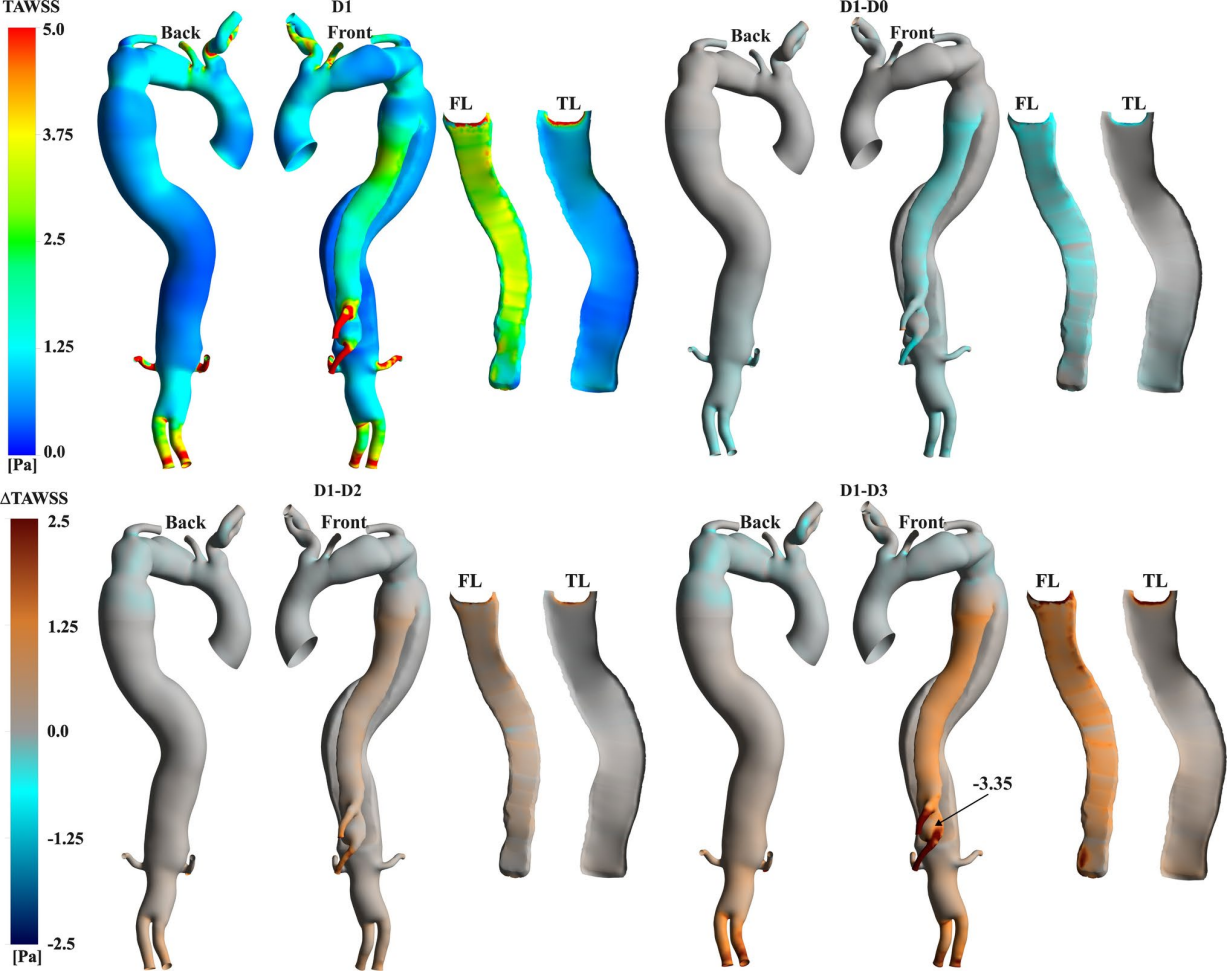
Contours of TAWSS for the patient-specific simulation (D1) and point-wise differences against additional cases.

**Fig. 8:**
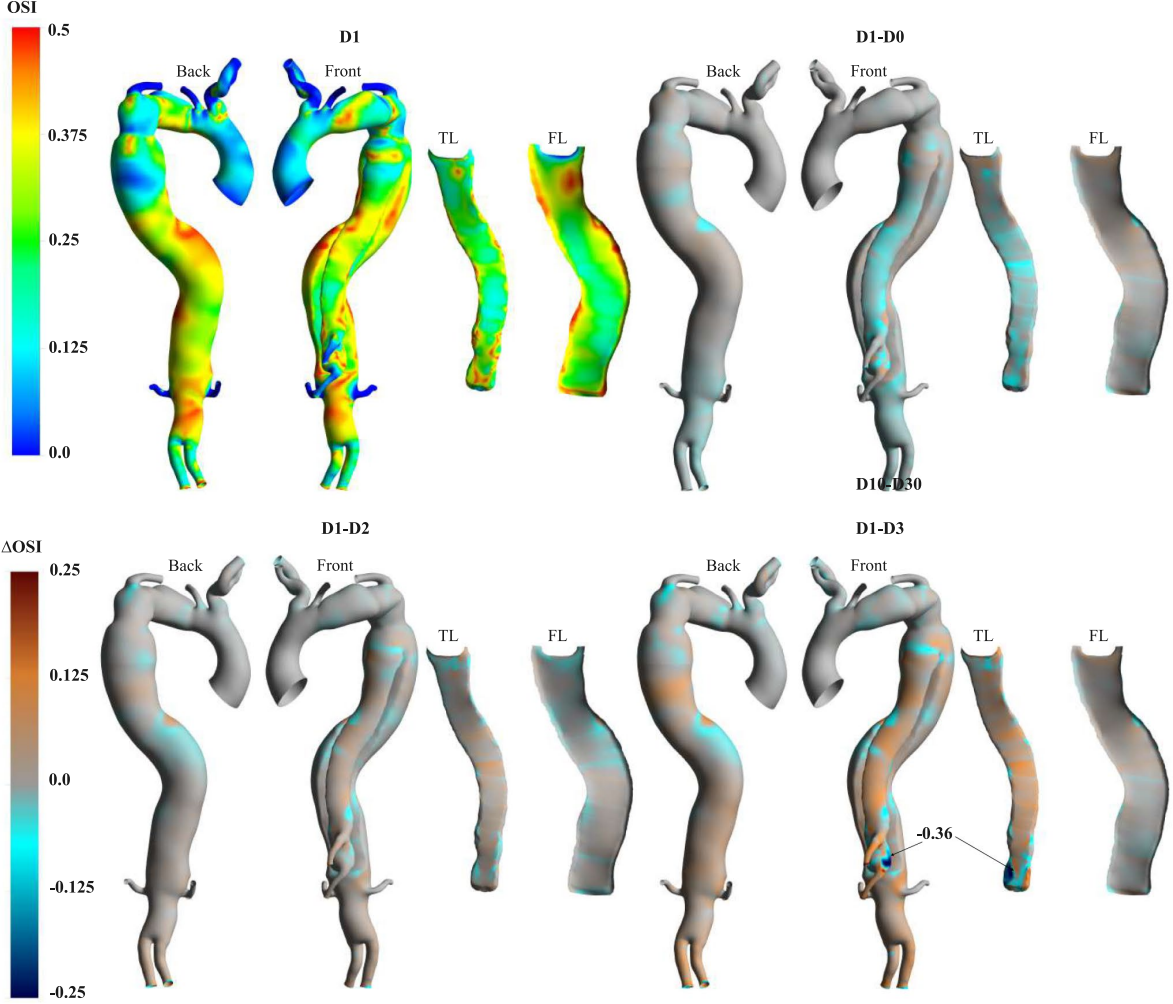
Contours of OSI for D1 and point-wise differences against additional cases

**Fig. 9:**
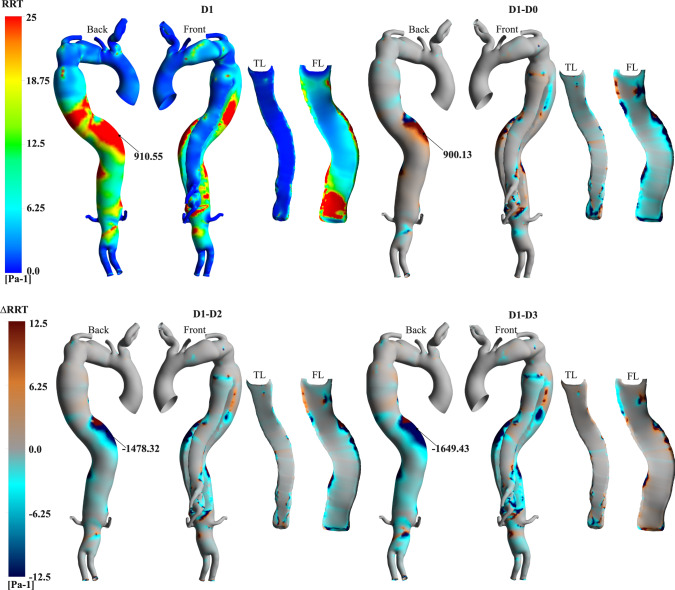
Contours of RRT for D1 and point-wise differences against additional cases.

Figures 7, 8, and 9 display the contours of time-average wall shear stress (TAWSS), oscillatory shear index (OSI), and relative residence time (RRT) obtained for the patient-specific case (D1), alongside point-wise differences of these metrics from the additional cases.

Figure 7 displays qualitatively similar TAWSS distributions. High values (>5 Pa) are found at the outlets and PET, where high velocities occur. Significant point-wise differences are observed between the cases at the TL, celiac trunk (CT), and superior mesenteric artery (SMA) locations. TAWSS values tend to be higher for D0 at the FL and lower for more mobile IF simulations, with the highest difference being − 3.35 Pa at the SMA compared to D3.

The highly fluctuating OSI indicates disturbed flow in both lumina in all cases (Fig. 8). Notably, regions of high OSI are present at the arch, along both lumina and proximal to the VAO. The D1-D0 point-wise differences highlight that the rigid IF simulation slightly underpredicts OSI values at the FL. Conversely, higher OSI values are predicted at the TL in D2 and D3. Additionally, proximal to the VAO at the bottom of the FL, where higher displacements occur, a − 0.36 point-wise difference is measured in the D1-D3 comparison (Fig. 9).

Relatively low TAWSS and fluctuating OSI lead to high RRT (>25 ${\text{Pa}}^{-{1}}$) at both lumina and the VAO in D1 (Fig. 9). Specifically, the highest RRT observed is 910.55 ${\text{Pa}}^{-1}$ at the DA. The rigid flap simulation does not capture this localised region of elevated RRT. Conversely, this high RRT region is accentuated in D2 and D3, with the maximum point-wise difference reaching − 1649.43 ${\text{Pa}}^{-{1}}$ in D3.

## Discussion

This study aimed to understand the impact of IF displacement in TBAD haemodynamics by further developing a previously presented moving boundary method to account for intimal flap motion. Our approach relies solely on MRI sequences acquired during a single session, including 4D Flow MRI, cine-MRI, and high-resolution TRUFI, along with brachial pressure measurements as input clinical data.

Quantifying IF mobility, a highly patient-specific variable, has been explored in various studies, revealing its crucial role in influencing malperfusion, haemodynamics, and treatment efficacy throughout different stages of TBAD [41, 42]. IF displacement is typically found to range from 0 to 3 mm in chronic cases [8].The patient-specific simulations (D1) revealed detailed insights into the interplay between intimal flap (IF) displacement, transmural pressure (TMP), and associated area changes throughout the cardiac cycle. These findings align with literature observations, demonstrating that these factors are associated with TL compression, FL expansion, and increased risk of rupture [43, 44]. The IF displacement reaches its maximum at peak systole (T1), resulting in TL compression and FL expansion due to high pressure within the FL (Figs. 4, 5). During the deceleration phase, these FL/TL compression/expansion patterns were inverted as the IF moved back towards its diastolic position, promoting a positive TMP and aligning with the reduction of cross-sectional area at the VAO (Fig. 6). Neglecting the motion of IF in numerical simulations of TBAD (D0) is associated with lower TMP at every time point in the cardiac cycle. This has potential clinical implications since it suggests that rigid IF simulations may lead to inaccurate luminal remodelling predictions, as a TMP close to zero is associated with better FL remodelling outcomes [9, 45]. Whilst the pressure differences are small, they can be attributed to the IF displacement, as D0 and D1 simulations share identical boundary conditions, inlet profiles, and geometry, isolating the effect to the compliant IF motion in D1. This highlights the sensitivity of local flow dynamics to small pressure variations driven by the flap mobility.

Conversely, simulations assuming a more compliant IF exhibited higher pressures within the FL during peak systole. This facilitated higher cross-sectional area variations, with the most significant expansions noted at the VAO for the D3 case. This suggests that a more mobile IF could contribute to higher TMP, possibly influencing luminal expansion and impacting flow conditions. The TMP trends observed align with previous studies (Fig. 4), which proposed a correlation between IF motion and risk of FL growth [23, 27]. This emphasises the potential of our model to further explore the mechanism of disease progression, particularly for highly mobile IF.

Compliant models, whether experimental or simulation based, have been shown to more accurately mimic aortic flow compared to 4D flow MRI, according to recent research [26, 46, 47]. Studies on TBAD showed that 4D flow MRI provides a good qualitative overview of flow patterns in regions of interest within the aorta. However, 4D flow MRI fails to accurately quantify flows in low-velocity and highly aneurysmal regions, where a poor signal-to-noise ratio (SNR) degrades flow measurement quality (Fig. 2) [48, 49]. CFD simulations informed by 4D flow MRI, as in the present study, offer an opportunity for detailed haemodynamic analyses [32, 50] even in regions that 4D flow MRI fails to capture, e.g. low flow velocity regions during diastole and near wall haemodynamics (Figs. 2, 3, 4, 5, 6, 7, 8, 9). Notably, using the three-dimensional inlet velocity at the inlet successfully replicated the velocity magnitude distribution in the AA as seen in the 4D flow MRI (Fig. 2).

In the proximal region to the IF, the patient-specific simulation (D1) exhibited good agreement with 4D flow MRI during the systolic time points (T0, T1, and T2). Notably, at the PET, DA, and VAO, the simulation accurately captured the high-velocity magnitudes within the TL, which were linked to elevated pressure and IF displacement, as well as the overall luminal velocity magnitude distribution. This is critical since it does not only validate the model, but also facilitate the evaluation of additional haemodynamic metrics essential for assessing the risk of aortic degeneration.

In contrast, the rigid IF simulation (D0) underpredicted velocity magnitudes in the TL and overpredicted them in the FL at the PET, DA, and VAO. It also showed poor agreement in terms of flow distributions, particularly during T1 and T2 (Fig. 2). These discrepancies were most pronounced in the proximal region of the IF, where limited displacement did not capture well the local haemodynamics. These findings underscore that it is important to incorporate IF movement in these simulations, as this plays an important role when calculating haemodynamic indices that attempt to predict patient-specific outcomes derived from these models.

At the visceral branches, higher flow rate differences were observed with a more mobile IF than *in vivo* measurements, with errors reaching up to 4.5%. This has implications for the potential prediction of malperfusion since a highly mobile IF can alter flow through the visceral branches.

The complex geometry of the aortic arch has been found to induce rotational flow and vortices, potentially contributing to the development of aortic dissection [51, 52]. Furthermore, the presence of rotational flow/vortical structures has been recently linked to the remodelling and growth of localised regions in both TBAD and reconstructed TBAD [32, 53]. Vortical structures were found to dominate the FL in a TBAD study [54]. These structures expanded and clustered around the entry tear during systole, causing frequent platelet collisions and likely promoting thrombus formation. Additionally, recent studies demonstrated that the intensity and the topology of helical flow structures can be affected when comparing compliant to rigid wall simulations [55, 56]. In our study, the vorticity contours at the PET in D1 highlighted the presence of counter-rotating vortices, leading to high IRF values (Fig. 6). The most complex patterns were observed at peak systole (T1). Such vortical patterns were less evident at the DA and VAO, where a separation between clockwise and anticlockwise vorticity was clear in both lumina. Swirling flows are not well captured when a rigid IF was assumed (D0) in regions where high IF displacements were predicted, for example, at the VAO during systole (T0, T1 and T2). Similar vorticity locations with higher magnitudes were simulated when the IF reached greater displacements in D2 and D3 (Fig. 6). At the PET and VAO, swirling patterns differed at peak systole. This local haemodynamics impact affected the WSS distributions as observed in Figures 7, 8, and 9 [57].

The IRF, which gives a measure of the intensity of the rotation of the flow on a plane, has been proposed as a marker of ascending aorta dilation [38, 58]. Additionally, in TBAD pre- and post-surgery studies, it was also indicated that reduced IRF can be linked to low WSS at the descending aorta, and, hence, the promotion of thrombosis and local growth [53, 59–62]. In line with these findings, our study demonstrated that the patient-specific three-dimensional inlet velocity used as an inlet boundary condition contributed to high circulation intensity at the AA. Moreover, our model provided a finer distinction between the TL and FL compared to the 4D flow MRI-based studies cited earlier, and the patient-specific simulation (D1) predicted elevated IRF values specifically within the TL at the PET and VAO, coinciding with vortical structures (Table 2). This suggests that under certain flow conditions, high IRF may not be confined to the AA but may also be present along the compressed lumen, in this case the FL, where patient-specific aortic morphology and flow dynamics contribute to increased rotational flow intensity. Conversely, as observed in the literature, IRF was reduced within the FL, potentially indicating an increased risk of local growth (Table 2).

Discrepancies in IRF values can be noted at the PET and VAO when comparing D0 with D1, demonstrating the shortcomings of using a rigid IF assumption in fully capturing the rotational nature of the flow. The increased IF mobility in D2 and D3 led to higher IRF magnitudes and a shift in flow direction within the false lumen (FL) at T1. These IRF values are induced by flow pattern changes, which can affect areas of stagnation and recirculation, impacting WSS distributions in the FL even after TEVAR. Such alterations in WSS may influence the risk of further vessel wall damage and negatively influencing patient outcomes.

Research has shown that IF motion impacts flow dynamics and pressures and plays a crucial role in thrombus formation dynamics [25]. Abnormal aortic haemodynamics have been shown to affect WSS distributions and associated metrics [13]. For example, colocation of low TAWSS and high OSI has been linked to aortic growth, thrombosis and high RRT (Fig. 7) [32, 63, 64]. Thus, high OSI values, triggered by the flow circulation close to the visceral branches, and low TAWSS in the distal and narrowed portion of the FL in D1 (Fig. 8) suggest the likelihood of cell deposition therein. Such conditions were also observed at the DA. More specifically, RRT > 900 Pa^-1^ values were predicted at the DA, coinciding with locations of chaotic flow and indicating an increased risk of aortic remodelling (Fig. 9). Similar observations can be made at the PET, where a high TAWSS > 5 Pa, due to a high-velocity and chaotic vortical structures forming at the luminal flow separation location, could promote a risk of aneurysmal formation or local wall rupture (Fig. 7). D0 did not replicate this pattern close to the SMA, suggesting that accounting for compliant IF simulations may be important for assessing markers related to the risk of aortic wall remodelling in TBAD.

The moving boundary method employed in this study makes certain simplifications, such as assuming a linear relationship between displacement and force. Due to limitations in the temporal resolution of 4D flow MRI and cine-MRI, obtaining a transient description of the discrete radial and non-elastic behaviour of the aorta was impossible. Additionally, since only one plane of cine-MRI captures the displacement of the IF, a constant stiffness had to be applied across the entire IF. Although the wall and intimal flap measurements taken from the cine-MRI fall within the resolution error margin of the imaging technique, the simulation results remained consistent with these measurements, demonstrating the validity of the approach. The method assumes that the pair of IF patches share the same normal, singular coordinate system, which should be considered for every node. This would be computationally heavy, and since the comparison of D1 against *in vivo* data showed good accuracy, the approach proposed here was deemed an acceptable compromise. Since this study included only a single post-TEVAR patient, further research will aim to collect higher-resolution in vivo data from a larger cohort, including patients with non-operated and post-open-surgery TBAD, to assess the transferability of the conclusions drawn in this study.

In conclusion, this study provided insights into the critical impact of IF mobility on TBAD haemodynamics using an improved moving boundary method informed by 4D flow MRI data. By appropriately capturing the patient-specific IF displacement, models revealed that increased IF mobility exacerbated TMP and impacted flow conditions, potentially leading to luminal expansion, thrombus formation, and aortic rupture. The findings underscored the importance of using compliant IF models over rigid simulations for accurate remodelling predictions and the impact of IRF, vorticity, and WSS on disease progression and treatment outcomes.

Clinically, these insights could inform more effective intervention strategies, such as tailored surgical planning to mitigate the adverse effects of a mobile IF and better understand whether or not a conservative approach might lead to optimal outcomes. Future research should focus on gathering higher-resolution *in vivo* data to refine these models further and enhance their clinical applicability.
